# 3D Spheroids of Umbilical Cord Blood MSC-Derived Schwann Cells Promote Peripheral Nerve Regeneration

**DOI:** 10.3389/fcell.2020.604946

**Published:** 2020-12-17

**Authors:** Yu-Jie Lin, Yun-Wei Lee, Che-Wei Chang, Chieh-Cheng Huang

**Affiliations:** ^1^Institute of Biomedical Engineering, National Tsing Hua University, Hsinchu, Taiwan; ^2^Department of Medical Science, National Tsing Hua University, Hsinchu, Taiwan

**Keywords:** peripheral nerve injury, regenerative medicine, 3D cell spheroids, umbilical cord blood mesenchymal stem cells, cell therapy

## Abstract

Schwann cells (SCs) are promising candidates for cell therapy due to their ability to promote peripheral nerve regeneration. However, SC-based therapies are hindered by the lack of a clinically renewable source of SCs. In this study, using a well-defined non-genetic approach, umbilical cord blood mesenchymal stem cells (cbMSCs), a clinically applicable cell type, were phenotypically, epigenetically, and functionally converted into SC-like cells (SCLCs) that stimulated effective sprouting of neuritic processes from neuronal cells. To further enhance their therapeutic capability, the cbMSC-derived SCLCs were assembled into three-dimensional (3D) cell spheroids by using a methylcellulose hydrogel system. The cell–cell and cell–extracellular matrix interactions were well-preserved within the formed 3D SCLC spheroids, and marked increases in neurotrophic, proangiogenic and anti-apoptotic factors were detected compared with cells that were harvested using conventional trypsin-based methods, demonstrating the superior advantage of SCLCs assembled into 3D spheroids. Transplantation of 3D SCLC spheroids into crush-injured rat sciatic nerves effectively promoted the recovery of motor function and enhanced nerve structure regeneration. In summary, by simply assembling cells into a 3D-spheroid conformation, the therapeutic potential of SCLCs derived from clinically available cbMSCs for promoting nerve regeneration was enhanced significantly. Thus, these cells hold great potential for translation to clinical applications for treating peripheral nerve injury.

## Introduction

Peripheral nerve injury, which is often caused by traumatic accidents and can lead to considerable long-term disability, is a worldwide clinical issue associated with significant socioeconomic burden (Hoke, 2006). Although intrinsic repair mechanisms can be initiated spontaneously, axonal regrowth is rather slow, which may ultimately result in a failure to reinnervate the target tissue (Zhang et al., 2017). Clinically, transplantation of autologous nerve to provide a physical scaffold with a proregenerative microenvironment for promoting axonal regrowth is considered the gold standard for treating peripheral nerve injury (Scheib and Hoke, 2013). However, several obstacles, including limited donor tissue availability, size/fascicle mismatch, painful neuroma formation, and a second surgery for tissue harvesting, restrict the use of nerve autografts (Schmidt and Leach, 2003; Deumens et al., 2010; Gu et al., 2014; Anderson et al., 2015). Therefore, alternative therapeutic approaches are urgently warranted.

Schwann cell (SC)-based therapies may hold great potential in promoting peripheral nerve repair. It has been reported that SCs can direct peripheral nerve repair by multiple mechanisms, including the secretion of neurotrophic factors, the establishment of regenerative templates, and the remyelination of demyelinated axons (Sowa et al., 2017). Therefore, transplantation of SCs has been demonstrated to be an effective strategy for enhancing neuron survival and promoting axon regrowth, thus achieving considerable therapeutic benefits (Sowa et al., 2017). Unfortunately, the clinical application of SC-based transplantation therapy has been hindered by the difficultly in harvesting a sufficient number and purity of functional SCs (Mazzara et al., 2017). To address this problem, various types of stem cells, including pluripotent stem cells (Kim et al., 2017), neural stem cells (Christopherson et al., 2009), mesenchymal stem cells (MSCs) (Xue et al., 2017), and adipose-derived stem cells (Tomita et al., 2013), have been investigated for their ability to transdifferentiate into SCs. Among these cell types, umbilical cord blood MSCs (cbMSCs) represent a clinically feasible candidate because they can be harvested autologously and non-invasively, cryopreserved for a long period, thawed and cultivated for rapid proliferation (Sung et al., 2012). Thus, the desired quantity of cells for transplantation therapy can be obtained without inducing adverse side effects. Furthermore, MSCs are known to possess efficient immunomodulatory activity and thus can engineer a proregenerative local microenvironment for accelerating tissue repair (Ballini et al., 2017, 2018; Spagnuolo et al., 2018), thereby further benefiting the regeneration of injured peripheral nerve.

In addition to the availability of an ideal cell source, the other fundamental issue faced by SC-based therapies for treating peripheral nerve injury is the retention and survival of the administered SCs in target area. To perform cell therapy, SCs must be collected and administered directly to the injured site. During cell harvesting, disruption of cell–cell interactions and cell–ECM connections is inevitable. However, for anchorage-dependent cells, these processes can lead to anoikis and significant cell death before transplantation (Koda et al., 2008; Robey et al., 2008; He et al., 2015; O'Neill et al., 2016; Amer et al., 2017), thus dramatically reducing their therapeutic potential and the consequent beneficial effects. Therefore, a strategy that does not destroy the cell–cell and cell–extracellular matrix (ECM) interactions and can maintain cellular anchorage during cell preparation would be advantageous to the immediate cell survival rate and the subsequent therapeutic efficacy.

To address the abovementioned issues, this study aims to transplant cbMSC-derived SC-like cells (SCLCs) raised using a non-genetic approach in the configuration of multicellular three-dimensional (3D) spheroid, which is known to recapitulate the *in vivo* physiological conditions (Lee et al., 2016; Kim et al., 2019) and improve the cell retention and survival after injection (Lee et al., 2011; Huang et al., 2014). As reported by our (Yu et al., 2020) and other groups (Zhang et al., 2016; Petrenko et al., 2017), the 3D cell spheroids possess enhanced intercellular interaction and well-preserved ECM and can be harvested without the use of proteolytic enzymes, thus benefiting the cellular engraftment and the ultimate therapeutic efficacy of cell therapy. Using a rat model of sciatic nerve crush injury (Sung et al., 2012; Zhang et al., 2017), we demonstrated that intraepineurial transplantation of 3D cbMSC-derived SCLC spheroids effectively promoted peripheral nerve regeneration and hindlimb motor functional recovery.

## Materials and Methods

### Cell Culture

Human cbMSCs with constitutive red fluorescence protein (RFP) expression and SH-SY5Y neuroblast-like cells were purchased from the Bioresource Collection and Research Center, Food Industry Research and Development Institute, Hsinchu, Taiwan (BCRC 60605) and from the American Type Culture Collection, Manassas, VA, USA (CRL-2266), respectively. The cbMSCs were cultured in α-minimum essential medium (αMEM; Thermo Fisher Scientific, Waltham, MA, USA) supplemented with 20% fetal bovine serum (FBS; GE Healthcare Bio-Sciences, Pittsburgh, PA, USA), 4 ng/mL basic fibroblast growth factor (bFGF; PeproTech, Rocky Hill, NJ, USA), 30 mg/mL hygromycin B, and 200 mg/mL geneticin (both from Thermo Fisher Scientific). SH-SY5Y cells were grown in Dulbecco's modified minimum essential medium (DMEM) supplemented with 10% FBS, 25 mM glutamine, 100 U/mL penicillin, and 100 μg/mL streptomycin (all from Thermo Fisher Scientific). To induce the differentiation of SH-SY5Y cells into a neuronal phenotype, cells were treated with 10 μM retinoic acid (RA; Sigma-Aldrich, St Louis, MO, USA) for 5 days before exchanging the media with DMEM containing 1% FBS, 10 μM RA, 25 mM glutamine, 100 U/mL penicillin, and 100 μg/mL streptomycin (Alhazzani et al., 2018).

### Differentiation of cbMSCs Into SCLCs

The differentiation of cbMSCs into the SC lineage was performed as previously describe (Zhang et al., 2017). The differentiation process was initiated by replacing the growth medium of cbMSCs with αMEM containing 1 mM β-mercaptoethanol (Sigma-Aldrich) for 24 h. Cells were then incubated for 72 h with medium containing 35 ng/mL RA. Subsequently, growth medium with RA, 5.7 mg/mL forskolin (Sigma-Aldrich), 10 ng/mL bFGF, 5 ng/mL platelet-derived growth factor-AA and 126 ng/mL glial growth factor-2 (all from PeproTech) was employed for the cultivation of cbMSCs for 2 weeks to establish SCLCs (Dezawa et al., 2001; Caddick et al., 2006; Brohlin et al., 2009).

### Preparation of 3D Spheroids of cbMSC-Derived SCLCs

Aqueous methylcellulose (MC; 12% by w/v; Sigma-Aldrich) was prepared in phosphate buffered saline (5 g/L) solution (Huang et al., 2014; Yu et al., 2020). Fifty microliters MC solution was applied into each well of a 96-well culture plate and incubated at 37°C for 30 min before adding 150 μL culture medium that contained 10,000 cbMSC-derived SCLCs. After a 24 h incubation, the thus-formed 3D SCLC spheroids were collected for the following applications.

### Immunofluorescence Staining

Cells or 3D cell spheroids were fixed in 4% paraformaldehyde, permeabilized with 0.1% Triton X-100 (both from Sigma-Aldrich), and incubated with 5% normal goat serum (Vector Laboratories, Burlingame, CA, USA) for 60 min. The primary antibodies against S100, SRY-related HMG-box (SOX)10, glial fibrillary acidic protein (GFAP), βIII tubulin, brain-derived neurotrophic factor (BDNF), vascular endothelial growth factor (VEGF), laminin or collagen (all from Abcam, Cambridge, MA, USA) were applied to the cells, and the cells were incubated at 4°C overnight. Negative controls were achieved by omitting the procedure of primary antibody incubation. The next day, the cells were washed and incubated with Alexa Fluor 488 or Alexa Fluor 633-conjugated secondary antibodies (Thermo Fisher Scientific) for 1 h at 37°C. The nuclei were counterstained with 4′,6-diamidino-2-phenylindole (DAPI; Thermo Fisher Scientific). The samples were observed under a fluorescence microscope (Carl Zeiss, Oberkochen, Germany) or a laser scanning confocal microscope (Carl Zeiss).

### Real-Time Quantitative Polymerase Chain Reaction (PCR)

Quantitative PCR was conducted to evaluate the gene expression profiles of cbMSCs or the derived SCLCs after 20 days of induction. Total RNA of the experimental cells was isolated using TRIzol Reagent (Thermo Fisher Scientific) followed by reverse-transcribed into complementary DNA (cDNA) using High Capacity Reverse Transcription Kit (Thermo Fisher Scientific) according to the manufacturer's instructions. Quantitative PCR was performed in triplicate using Power SYBR Green PCR Master Mix (Thermo Fisher Scientific) in the StepOnePlus Real-Time PCR System (Thermo Fisher Scientific). The primer sequences are listed in Supplementary Table 1. The expression levels of *S100B, GFAP*, and *BDNF* were determined and normalized to housekeeping gene *GAPDH* expression. Data were pooled from three independent batches of differentiation.

### Effects of SCLCs on SH-SY5Y Cell Neurite Formation

The effects of cbMSC-derived SCLCs on the formation of neurites by SH-SY5Y cells were investigated using both indirect and direct coculture methods. For indirect coculture, the differentiated SH-SY5Y cells and SCLCs were inoculated into different wells in a μ-Slide 2 Well Co-Culture (ibidi GmbH, Munich, Germany). Although the two types of cells were grown individually, they shared the same medium during culture and could communicate by soluble factors and proteins. For direct coculture, the differentiated SH-SY5Y cells and SCLCs were mixed and plated into a μ-Dish (ibidi) for cultivation. All cells were grown in DMEM with 1% FBS, 10 μM RA, 25 mM glutamine, 100 U/mL penicillin, and 100 μg/mL streptomycin (Gibb et al., 2015). After coculture, the cells were fixed and processed for immunofluorescence staining. Amounts of neurites and the longest neurite length for each neuron in three random views were estimated using the US National Institutes of Health ImageJ software with NeurphologyJ plugin as described previously (Ho et al., 2011; Sowa et al., 2017; Chen et al., 2020).

### Rat Model of Sciatic Nerve Crush Injury and Cell Transplantation

All animal experiments were approved by the Institutional Animal Care & Utilization Committee, National Tsing Hua University, Hsinchu, Taiwan, and the care of the animals was in accordance with the Guidebook for the Care and Use of Laboratory Animals (third edition), published by the Chinese-Taipei Society of Laboratory Animal Sciences in 2000. Male Sprague-Dawley rats at 8 weeks of age were used for all experiments. For surgery, rats were anesthetized with 2% isoflurane and analgesia perioperatively with carprofen (5 mg/kg, administered subcutaneously). An incision was made on the right thigh posteriorly, and the biceps femoris muscle was bluntly dissected to expose the sciatic nerve. A type 5 watchmaker forceps was employed to crush the sciatic nerve for 30 s at a location 1 mm distal to the sciatic nerve bifurcation (Sung et al., 2012; Tomita et al., 2013; Zhang et al., 2017). Immediately after establishment of the crush injury, saline, cell suspensions of undifferentiated cbMSCs or cbMSC-derived SCLCs, or 3D spheroids of cbMSC-derived SCLCs were transplanted into the injured site using a Hamilton syringe with a 26-gauge needle (5 × 10^5^ cells in 20 μL saline). Postoperative pain management was performed by administering carprofen (5 mg/kg, subcutaneous) once daily for 3 days. Immunosuppression using cyclosporine A (Sandimmune, Novartis, Basel, Switzerland; 25 mg/kg/day) was delivered subcutaneously 3 days before cell transplantation and continued daily until animals were euthanized.

### Gait Analysis

Gait analysis was conducted to assess motor function before injury and at 1–6 weeks postoperatively (Sowa et al., 2017; Rodríguez Sánchez et al., 2019). The hindpaws of test animals were dipped in ink, and the rats were allowed to walk down the paper-covered track, leaving their hindpaw ink prints for recording. Three animals were recruited per experimental group, and each animal was allowed to run along the track for three times per time point. The sciatic function index (SFI) was calculated using the following equation: SFI = −38.3 (EPL – NPL)/NPL + 109.5 (ETS – NTS)/NTS + 13.3 (EIT – NIT)/NIT – 8.8 (Hsu et al., 2019). The print length (PL) represents the longitudinal distance from the heel to the tip of the third toe, the toe spread (TS) refers to the distance between the first and fifth toes, and the intermediary toe spread (IT) represents the distance between the second and fourth toes. EPL, ETS, and EIT are recorded from the experimental (E) foot, while NPL, NTS and NIT are from the normal (N) foot.

### Histological Analysis

At 1 h or 6 weeks postsurgery, the rats were anesthetized, and their operated sciatic nerves were excised and fixed in 10% phosphate-buffered formalin, cryoprotected in 20% sucrose, and embedded in optimum cutting temperature compound (Surgipath FSC22, USA). Longitudinal cryosections were sliced into 7-μm thick sections using a cryostat microtome (CM1950, Leica, Wetzlar, Germany), and immunostaining was performed as described above.

### Statistical Analysis

Data are expressed as the mean ± standard deviation. Statistical analyses were performed using GraphPad Prism software (version 8.2; San Diego, CA, USA). For the comparison of two groups, an unpaired, two-tailed Student's *t*-test was used. One-way ANOVA with Tukey's correction was employed for the comparison of three or more groups. Differences are considered to be significant at *P* < 0.05.

## Results

### Conversion of cbMSCs into SCLCs Using a Non-genetic Approach

**Figure 1:**
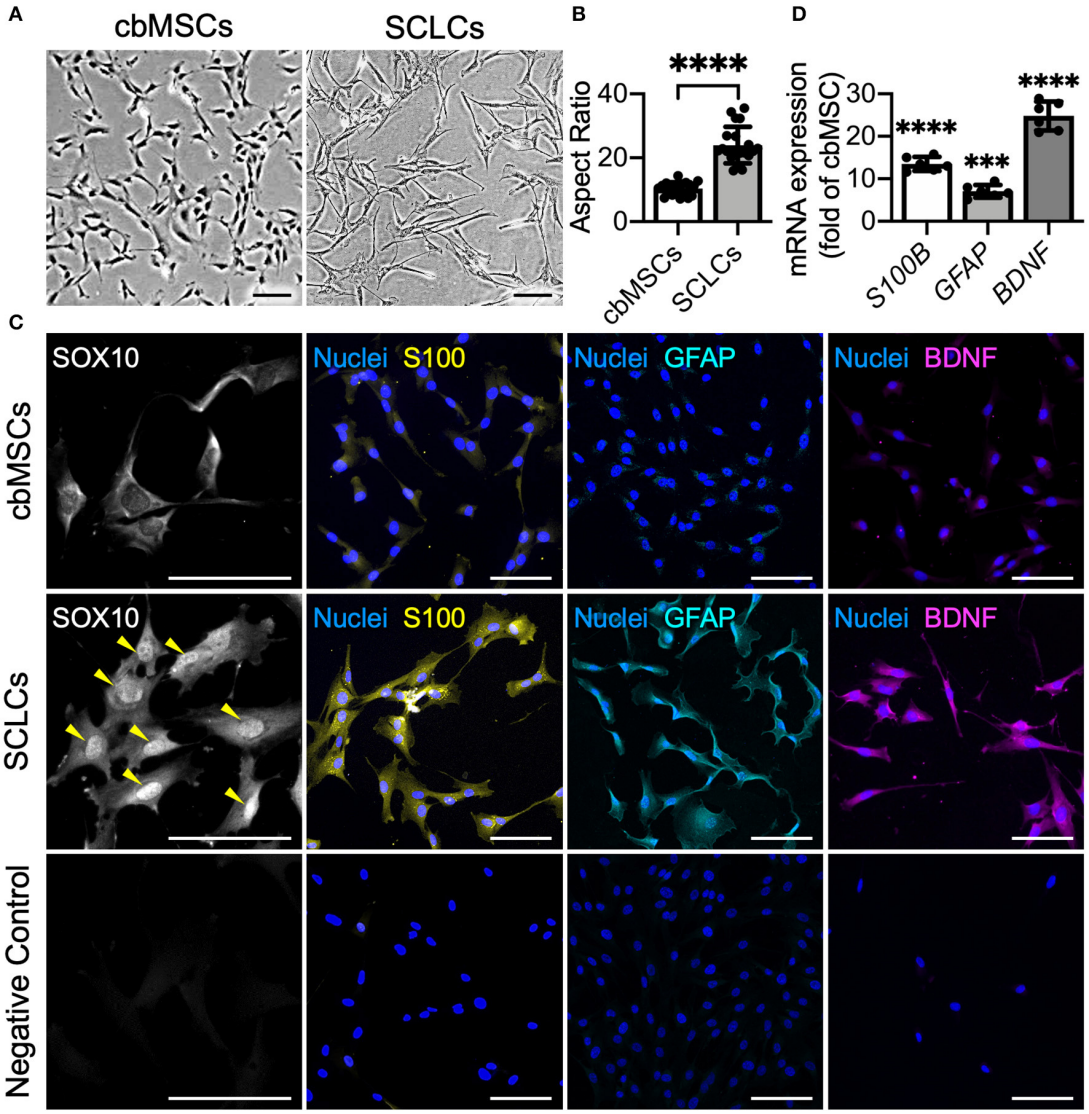
Umbilical cord blood mesenchymal stem cells (cbMSCs) are converted into Schwann cell (SC)-like cells (SCLCs) using a non-genetic approach. **(A)** Morphological characteristics of the cbMSCs before and after being converted into SCLCs and **(B)** their corresponding aspect ratios (*n* = 20 cells pooled from 2 independent experiments, mean ± s.d.) ****P < 0.001. **(C)** Expression of SC markers SOX10, S100, and GFAP and neurotrophic factor BDNF by cbMSCs and cbMSC-derived SCLCs detected by immunofluorescence staining or **(D)** determined by real-time quantitative polymerase chain reaction. The results are expressed as the fold change relative to the undifferentiated cbMSCs (*n* = 6 pooled from 3 independent batches of differentiation, mean ± s.d.). Samples immunostained without primary antibodies were used as negative controls. Arrow heads indicate nuclear accumulation of SOX10. Scale bars: 100 μm. ***P < 0.005 vs. undifferentiated cbMSCs. ****P < 0.001 vs. undifferentiated cbMSCs.

As indicated by phase-contrast micrographs, naïve cbMSCs had a spindle cell shape, whereas after induction by using defined chemicals, the differentiated cbMSCs exhibited a significantly elongated morphology (Figure 1A). The aspect ratio (cellular length *vs*. width) of the differentiated cbMSCs (22.5 ± 7.3) increased significantly compared to that of the undifferentiated cbMSCs (10.4 ± 2.1; *P* < 0.001; Figure 1B). We then evaluated the expression of SC-related markers by cbMSCs after induction. The results of immunofluorescence staining showed obvious nuclear accumulation of SOX10 at 7 days after the initiation of induction (Figure 1C). After exposing to induction medium for 20 days, the cbMSCs exhibited significantly upregulated expression of SC markers S100 and GFAP compared with that under regular culture conditions (Figure 1C). Additionally, an increased level of BDNF was observed in SCLCs (Figure 1C). The results of real-time quantitative PCR further confirmed the expression of multiple SC markers in the harvested SCLCs (Figure 1D).

**Figure 2:**
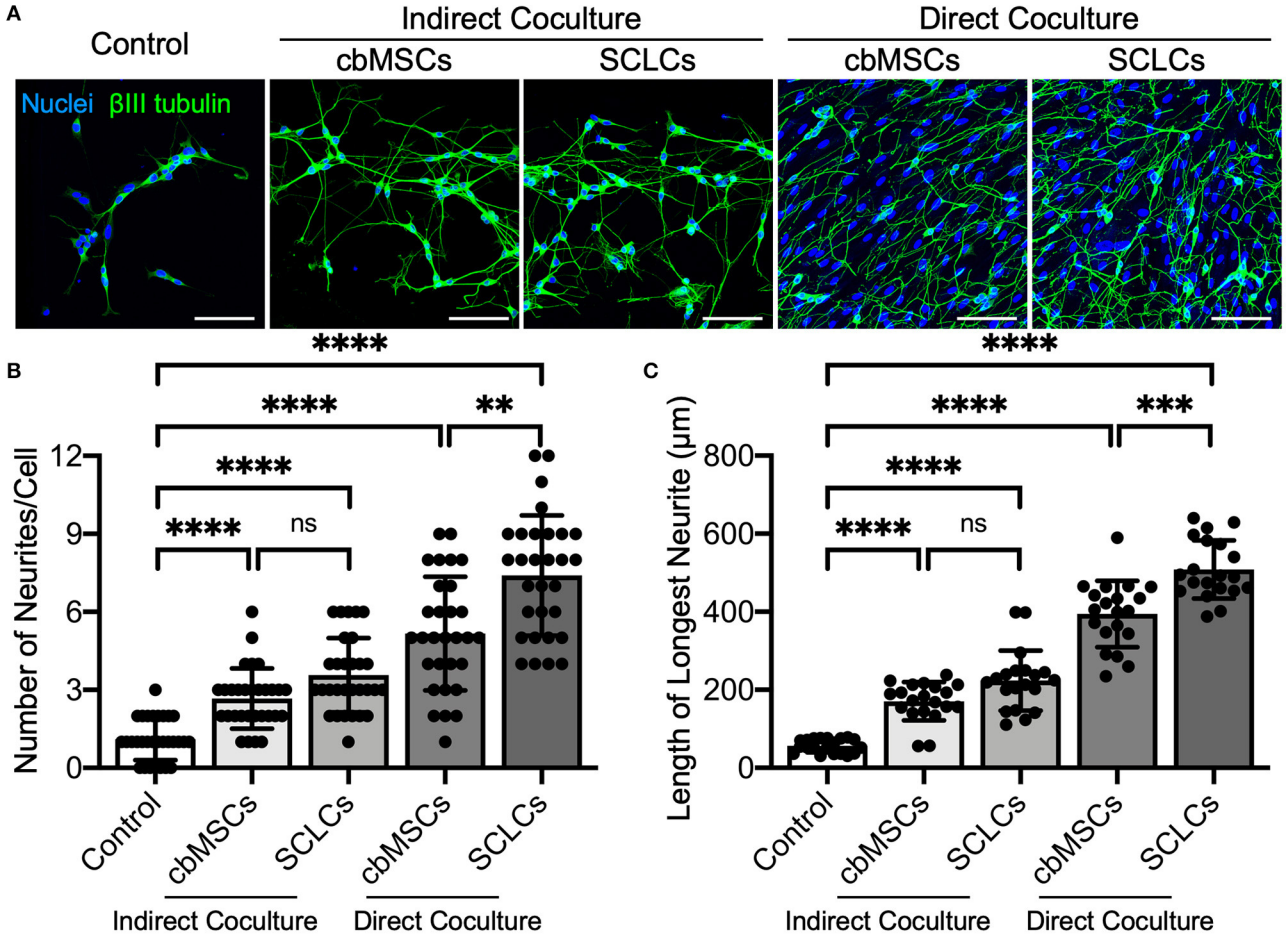
The cbMSC-derived SCLCs promote neurite process sprouting. **(A)** Coculture of SCLCs with SH-SY5Y neuronal-like cells significantly promoted neurite formation, as indicated by the fluorescence images of neurite-specific protein βIII tubulin. **(B)** The number of neurites per neuron (n = 30 cells pooled from 2 independent experiments) and **(C)** the length of the longest neurite of each cell (*n* = 20 cells pooled from 2 independent experiments) were estimated (mean ± s.d.). Scale bars: 100 μm. **P < 0.01; ***P < 0.005; ****P < 0.001; ns, not significant.

As a functional evaluation, we examined whether the obtained SCLCs could promote neurite outgrowth of neurons. Undifferentiated cbMSCs and cbMSC-derived SCLCs were directly or indirectly cocultured with SH-SY5Y cells. The neurites were visualized by immunofluorescent labeling of βIII tubulin to estimate their number and length. As shown in Figure 2A, treatment with conditioned media derived from cbMSCs or SCLCs (indirect coculture) slightly promoted neurite formation of SH-SY5Y cells. Conversely, significantly enhanced neurite outgrowth of SH-SY5Y cells was observed when cells were directly cocultivated with cbMSCs or SCLCs. Statistical analyses of neurite structures confirmed the superiority of SCLCs over undifferentiated cbMSCs by revealing significantly more neurite branching (Figure 2B; *P* < 0.001) and longer neurite extension (Figure 2C; *P* < 0.005) of SH-SY5Y cells.

### SCLCs Exhibit Enhanced Therapeutic Potential After Assembling Into 3D Spheroid

**Figure 3:**
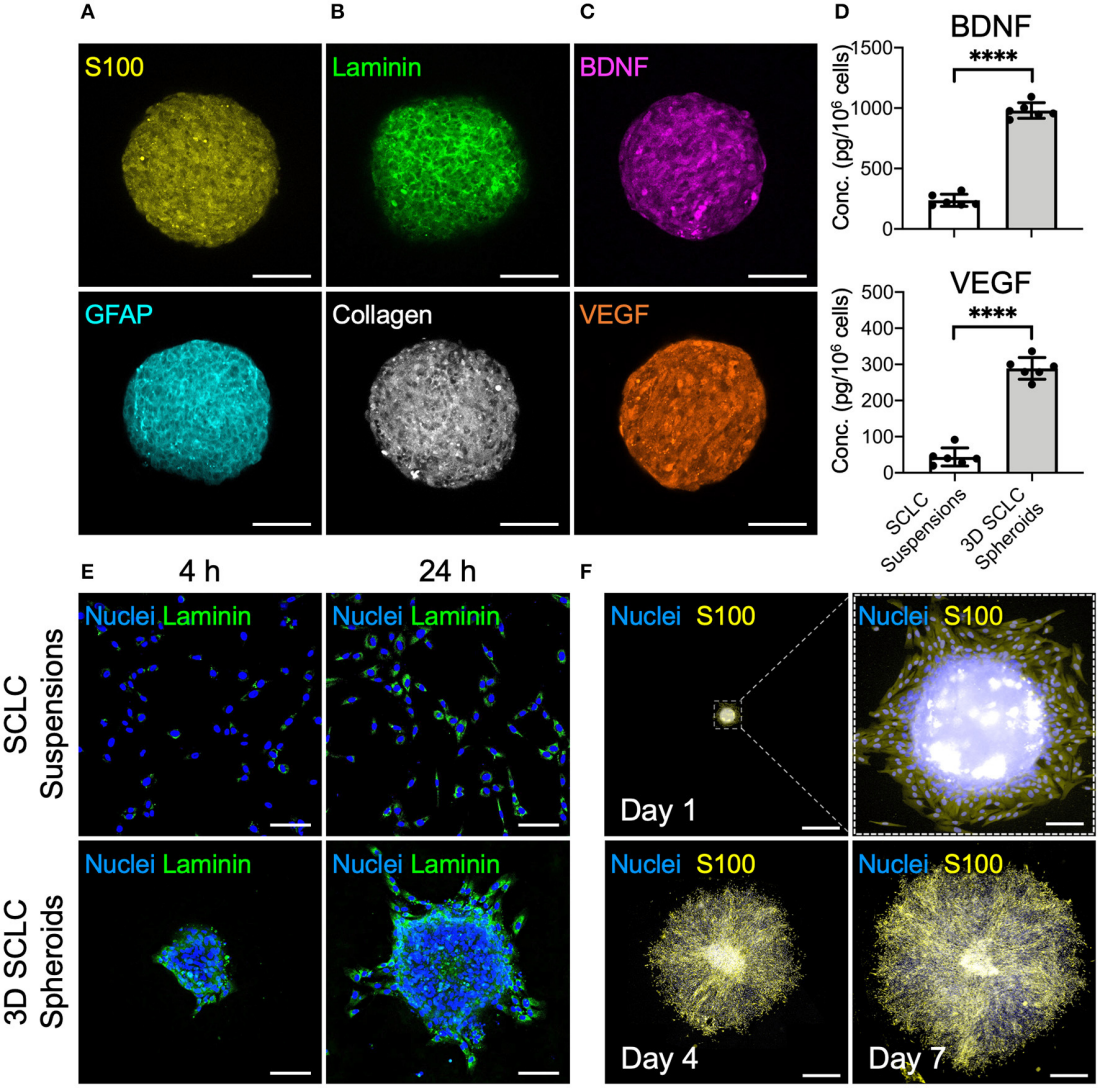
SCLCs were assembled into three-dimensional (3D) spheroids to enhance their therapeutic potential. Immunofluorescence evaluation of the expression of **(A)** SC markers S100 and GFAP, **(B)** extracellular matrix proteins laminin and collagen, and **(C)** neurotrophic factor BDNF and proangiogenic factor VEGF. Scale bars: 100 μm. **(D)** The concentrations of BDNF and VEGF in the collected 3D SCLC spheroids or SCLC suspensions obtained using conventional trypsinization were determined by ELISA (*n* = 6, mean ± s.d.). **(E)** The attachment and proliferation of 3D SCLC spheroids or SCLC suspensions that were harvested and transferred to culture plates were evaluated by using immunofluorescence staining. Scale bars: 100 μm. **(F)** The spreading of 3D SCLC spheroids over culture plate as time progressed. Scale bars: 1 mm; scale bar in zoom box: 100 μm. ****p < 0.001.

The cbMSC-derived SCLC suspensions were cultivated in the MC hydrogel-coated plate to fabricate 3D cell spheroids. Evaluation of the 3D SCLC spheroid diameters revealed a homogeneous size distribution with an average diameter of 245.8 ± 12.3 μm (*n* = 4 batches). Immunostaining showed significant expression of the SC markers S100 and GFAP by the SCLCs after assembly into 3D spheroids (Figure 3A). As an ordinary enzyme-based approach was not employed to detach and harvest cells during preparation, abundant ECM proteins, such as laminin and collagen, were well-retained within the 3D cell spheroids (Figure 3B). Moreover, the immunofluorescence images in Figure 3C show that secretion of the neurotrophic factor BDNF and the proangiogenic factor VEGF were remarkably enhanced in the 3D spheroids of SCLCs after collection compared with their counterparts that were cultivated in a two-dimensional conformation and harvested using the conventional trypsinization method. These observations were further supported by the results of enzyme-linked immunosorbent assay (ELISA; Figure 3D; *P* < 0.001).

For cell-based therapy, the adhesion of the engrafted cells is a prerequisite for their survival and the subsequent therapeutic benefit. Therefore, we next investigated the cell adhesion capability of SCLC suspensions and 3D SCLC spheroids. After plating in a tissue culture polystyrene dish, the 3D SCLC spheroids with an inherent laminin meshwork were able to adhere to the plate surface within a short period (Figure 3E). Therefore, swift attachment and migration of the SCLCs from the 3D spheroids was observed. In contrast, SCLCs that were harvested by trypsinization took a longer period of time to attach to the culture plate (Figure 3E). Moreover, prompt proliferation and spreading of the SCLCs from the 3D spheroids over the culture plate was observed (Figure 3F).

### Transplantation of 3D SCLC Spheroids Promotes Functional and Structural Recovery of Rat Sciatic Nerve After Crush Injury

**Figure 4:**
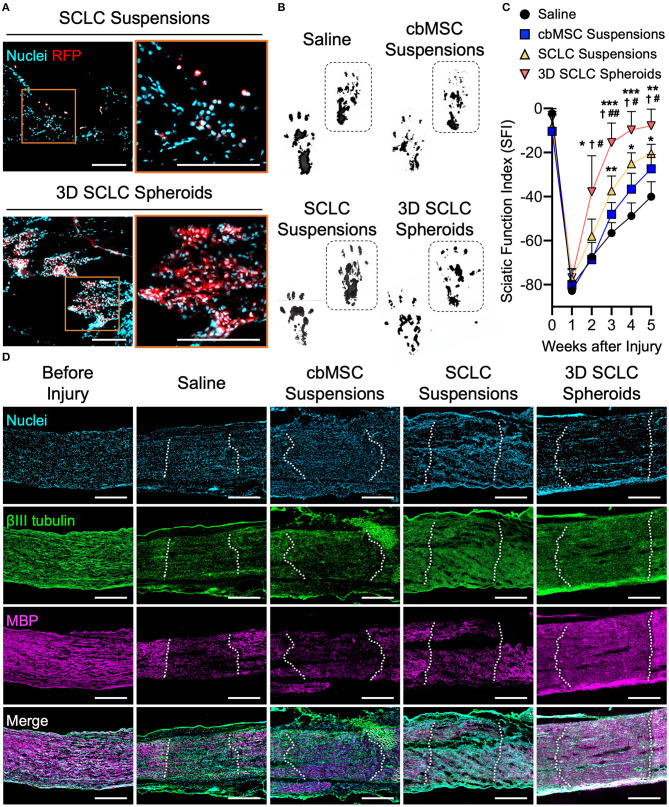
3D SCLC spheroids were transplanted into crush-injured rat sciatic nerves to promote regeneration. **(A)** Representative fluorescent images showing the retention of transplanted cells labeled with red fluorescence protein (RFP) in sciatic nerve tissues at 1 h postoperation. Orange box is enlarged in right panel. Scale bars: 200 μm. **(B)** Representative hind feet tracks of mice recorded 4 weeks after cell transplantation and **(C)** the corresponding sciatic function index (SFI). The dashed boxes highlight the prints of the experimental feet (*n* = 3, mean ± s.d.). *P < 0.05 *vs*. saline group; **P < 0.01 vs. saline group; ***P < 0.005 vs. saline group; *P* < 0.05 vs. cbMSC-suspension group; ^#^*P* < 0.05 vs. SCLC-suspension group; ^##^*P* < 0.01 vs. SCLC-suspension group. **(D)** Expression of βIII tubulin and myelin basic protein (MBP) determined by immunofluorescence staining. The dotted lines indicate the borders of injury sites. Scale bars: 500 μm.

The SCLC spheroids were next transplanted into crush-injured sciatic nerves in a rat model. We first harvested the nerve tissues at 1 h after receiving SCLC spheroids or SCLC suspensions to track the engrafted cells that expressed RFP under a fluorescence microscope. When delivered as cell suspensions, the RFP-positive cells, indicating the engrafted SCLCs, were rarely detected and scattered in the tissue (Figure 4A). Conversely, a large population of RFP-labeled cells that were delivered with a 3D spheroid configuration were presented at the target area (Figure 4A), demonstrating the superior post-engrafted cell retention efficiency of 3D SCLC spheroids than that of SCLC suspensions.

Next, functional recovery of the injured sciatic nerve was evaluated by rat gait analysis. Animals that received saline, undifferentiated cbMSC suspensions or SCLC suspensions served as controls. Representative walking footprints of rats from each group at 4 weeks after cell engraftment are shown in Figure 4B. At baseline, walking track analysis revealed no statistically significant variation in the SFI among all investigated groups (*P* > 0.05; Figure 4C), suggesting that the degrees of impairment of hindlimb function in all test rats following surgically induced sciatic nerve crush injury were comparable. A clear improvement in the SFI began to appear in the SCLC spheroid group 3 weeks after cell engraftment. The SCLC spheroid group exhibited a significant (*P* < 0.05) improvement in the SFI (−7.9 ± 7.4) compared with the saline (−40.1 ± 6.8), cbMSC suspension (−27.4 ± 6.5), and SCLC suspension (−20.5 ± 4.2) groups at 5 weeks after cell transplantation (Figure 4C). Finally, the capability of 3D spheroids of cbMSC-derived SCLCs to promote structural recovery of the peripheral nerve was assessed. Histological data showed that the group that received transplantation of 3D SCLC spheroids exhibited more pronounced expression of myelin basic protein in the injured sciatic nerve than the other groups, suggesting enhanced remyelination (Figure 4D).

## Discussion

Herein, cbMSCs, a clinically applicable cell source, were converted into SCLCs followed by assembling into the 3D spheroid configuration for treating peripheral nerve injury. Using a non-genetic method reported in the literature (Zhang et al., 2017), accumulation of SOX10 protein, the crucial transcription factor for SC differentiation, in cell nuclei was observed in the induced cbMSCs. As time progressed, the increased expression of S100, GFAP and BDNF was observed. SOX10 is known as a marker that expressed constitutively during the entire SC development process, while S100, one of the most commonly used SC markers, is not expressed in Schwann cell precursor stage (Jessen and Mirsky, 2005; Liu et al., 2015). Therefore, the gradual elevation of S100 level indicated the differentiation of cbMSCs and the maturation of SCLCs. Morphologically, in consistent with typical SCs, the cbMSC-derived SCLCs possessed an elongated shape and had a high aspect ratio (Kaewkhaw et al., 2011; Tomita et al., 2013; Sowa et al., 2017).

To evaluate whether the cbMSC-derived SCLCs could functionally support neurite formation, they were employed to directly or indirectly coculture with SH-SY5Y cells, a neuronal-like human cell line (Gibb et al., 2015). It has been reported that after induction with RA, the SH-SY5Y cells can sprout neurite-like processes (Racchetti et al., 2010), and thus can be used as a model to evaluate the potential of cbMSC-derived SCLCs in promoting axonal regrowth. In the indirect coculture scenario, the conditioned medium derived from cbMSCs or SCLCs effectively enhanced the neurite formation. MSCs have been explored as efficient cellular and microenvironmental modulators in regenerative medicine owing to their potential in secreting numerous bioactive molecules (Caplan and Correa, 2011), including growth factors that can enhance neurite formation (Lee et al., 2017). Additionally, paracrine signaling is one of the major mechanisms that SCs exploit to promote axonal regeneration. In the direct coculture experiment, both cbMSCs and SCLCs exhibited a further increased potential in promoting neurite sprouting, especially the SCLC group. As the native SCs have close and complex interactions with axons (Wilson et al., 2020), it is not surprise that the direct interaction between SCLCs and SH-SY5Y cells could achieved the best result in stimulating neurite formation and elongation. Collectively, our results clearly demonstrated that cbMSCs can be phenotypically, epigenetically, and functionally converted into SCLCs using a well-defined non-genetic approach. More importantly, the obtained cbMSC-derived SCLCs stimulated the sprouting of neuritic processes from neuronal cells, suggesting that they are advantageous to the peripheral nerve microenvironment for promoting regeneration.

Therapeutic cells that are delivered in a 3D spheroid configuration have been demonstrated to have a higher therapeutic potential (Huang et al., 2015; Yu et al., 2020). It has been demonstrated that cell transplantation using a multicellular 3D spheroid conformation can achieve a better therapeutic outcome than using cell suspension, as cell–cell and cell–ECM interactions are completely preserved during implantation (Chen et al., 2013; Huang et al., 2014). In the present study, the SCLCs were assembled into 3D spheroids by using a MC hydrogel system. We previously reported that the MC hydrogel can provide a non-adhesive surface in the culture plate (Wang et al., 2009). Anchorage-dependent cells, such as SCs, need a surface or culture support for attachment and proliferation. As a result, the SCLCs seeded into the MC hydrogel-coated plate adhered to each other and thus assembled into 3D spheroids, which could be collected simply by using a Pipetman micropipette and transferred to a syringe for transplantation.

As ECM has been demonstrated to serve as an efficient reservoir for growth factors and cytokines that are deposited from cells (Ishihara et al., 2018), it is not surprising that much more paracrine factors, which play important roles in nerve regeneration, were retained in the 3D SCLC spheroids than in trypsin-dissociated cells. The aforementioned results strongly suggest that the assembly of SCLCs into a 3D spheroid conformation might offer additional benefits for the enhanced capability of paracrine factor secretion and preservation. Furthermore, the cellular attachment following administration, a fundamental criterion for the subsequent cell survival and exertion of therapeutic effects, could be accelerated significantly both *in vitro* and *in vivo* by introducing the 3D spheroid conformation, probably owing to the preservation of ECM with cell spheroids. Taken together, transplantation of cbMSC-derived SCLCs using a 3D cell spheroid conformation containing cells, ECM, and secreted growth factors provided a superior therapeutic effect than the transplantation of a simple cell suspension, which was harvested using the conventional trypsinization method and containing only cells but no deposited matrix or growth factors.

While we successfully developed 3D SCLC spheroids with enhanced therapeutic potential, there is still room for further improvement to accelerate the translational application. For example, animal serum that was employed for cultivating human cells in this study is known to be associated with significant safety issues and thus is not clinically applicable. Therefore, the development of xeno-free culture medium, such as chemically defined medium, human blood-derived serum, plasma, and platelet lysate (Marrazzo et al., 2016; Cimino et al., 2017), and surfaces/coatings/scaffolds (Jung et al., 2010; Marrelli et al., 2016) that can efficiently promote the propagation of MSCs and control their differentiation is highly warranted to realize the clinical application of the developed cell-based therapies.

In summary, the present findings demonstrate the feasibility of using cbMSC-derived SCLC as an alternative cell source for SC-based therapy to promote peripheral nerve regeneration. Moreover, by assembling SCLCs into a 3D-spheroid conformation, their therapeutic potential can be significantly enhanced, owing to the enhanced cell–cell and cell–extracellular matrix interactions. Such an approach may have great potential for translation to clinical applications for treating peripheral nerve injury.

## Data Availability Statement

The raw data supporting the conclusions of this article will be made available by the authors, without undue reservation.

## Ethics Statement

All animal experiments were approved by the Institutional Animal Care & Utilization Committee of National Tsing Hua University under the protocol number 10641.

## Author Contributions

Y-JL and C-CH conceived and designed the experiments, and wrote the manuscript. Y-JL, Y-WL, and C-WC carried out experiments and analyzed the data. All authors contributed to the article and approved the final version of the manuscript.

## Conflict of Interest

The authors declare that the research was conducted in the absence of any commercial or financial relationships that could be construed as a potential conflict of interest.
