# Supplementary material for: 3D Spheroids of Umbilical Cord Blood MSC-Derived Schwann Cells Promote Peripheral Nerve Regeneration
Source: Front Cell Dev Biol. 2020 Dec 17;8:604946. doi: 10.3389/fcell.2020.604946 (PMC7773632; doi:10.3389/fcell.2020.604946)
Supplement: Supplementary file 1 [file Table_1.DOCX]

3D Spheroids of Umbilical Cord Blood MSC-derived
Schwann Cells Promote Peripheral Nerve Regeneration

Yu-Jie Lin^1^, Yun-Wei Lee^1^, Che-Wei Chang^1,2^, Chieh-Cheng Huang^1*^

^1^ Institute of Biomedical Engineering, National Tsing Hua University, Hsinchu, Taiwan
^2^ Department of Medical Science, National Tsing Hua University, Hsinchu, Taiwan

**Table S1**. Primer sequence used for real-time quantitative polymerase chain reaction.

| Gene | Forward | Reverse |
| --- | --- | --- |
| *GAPDH* | 5’-AATCCCATCACCATCTTCCA-3’ | 5’-TGGACTCCACGACGTACTCA-3’ |
| *S100B* | 5’-GGAAATCAAAGAGCAGGAGGT-3’ | 5’-ATTAGCTACAACACGGCTGGA-3’ |
| *GFAP* | 5’-GTCCATGTGGAGCTTGACG-3’ | 5’-CATTGAGCAGGTCCTGGTAC-3’ |
| *BDNF* | 5’-CACTTGAGTCTCCAGGACAGC-3’ | 5’-ATCCAACAGCTCTTCTATCACG-3’ |
